# Supplementary material for: High-resolution temporal profiling of E. coli transcriptional response
Source: Nat Commun. 2023 Nov 22;14:7606. doi: 10.1038/s41467-023-43173-7 (PMC10665441; doi:10.1038/s41467-023-43173-7)
Supplement: Supplementary file 1 — Supplementary Information [file 41467_2023_43173_MOESM1_ESM.pdf]

High-Resolution Temporal Profiling of E. coli Transcriptional Response

Supplementary Information

Arianna Miano[1], Kevin Rychel[1], Andrew Lezia[1], Anand Sastry[1], Bernhard Palsson[1,2], Jeff Hasty[1,3]

[1]Department of Bioengineering, University of California San Diego, 9500 Gliman Dr, La Jolla, California, USA.
[2]Department of Pediatrics, University of California San Diego, 9500 Gliman Dr, La Jolla, California, USA.
[3]Division of Biological Science, University of California San Diego, 9500 Gliman Dr, La Jolla, California, USA.

Contributing authors: armiano@ucsd.edu; krychel@ucsd.edu; alezia@ucsd.edu; avsastry@ucsd.edu; bpalsson@ucsd.edu; jhasty@eng.ucsd.edu;

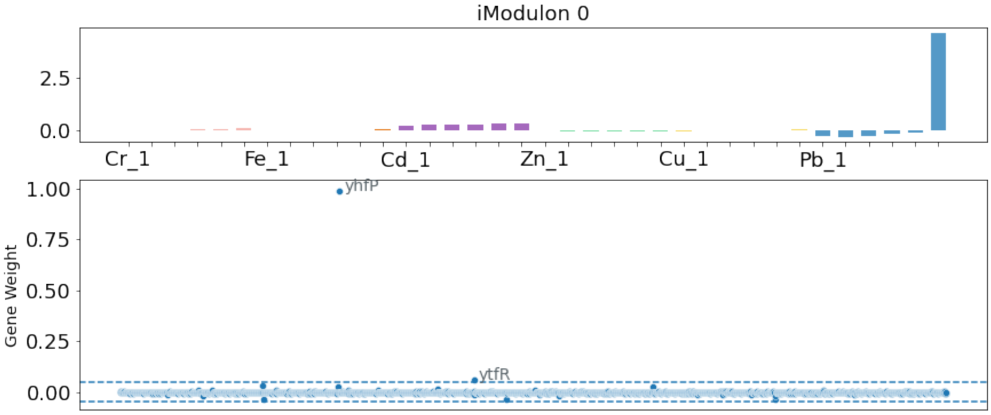

Fig S1|ICA results from running the algorithm on the induction window time points. Top: Activation profile plot of iModulon 0. Bottom: Genes weights above threshold for IModulon 0.

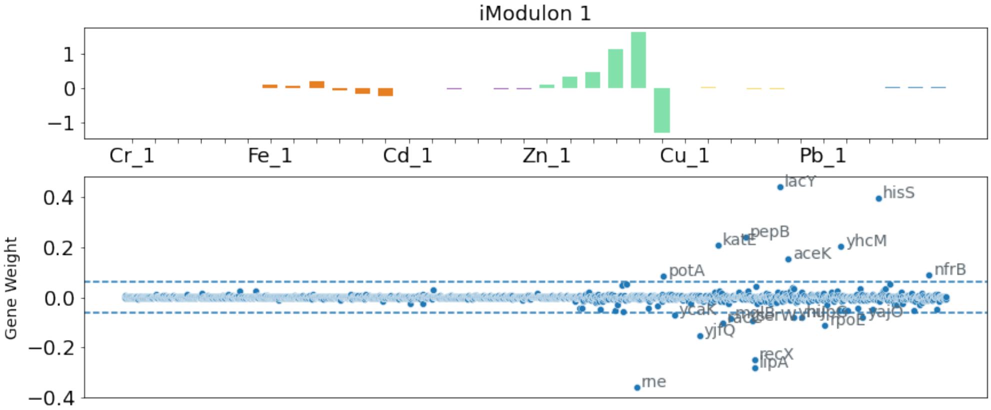

Fig S2|ICA results from running the algorithm on the induction window time points. Top: Activation profile plot of iModulon 1. Bottom: Genes weights above threshold for IModulon 1.

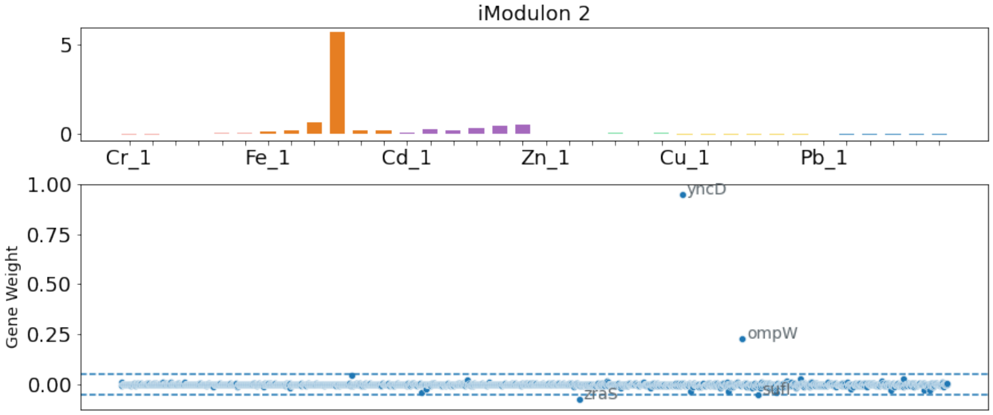

Fig S3|ICA results from running the algorithm on the induction window time points. Top: Activation profile plot of iModulon 2. Bottom: Genes weights above threshold for IModulon 2.

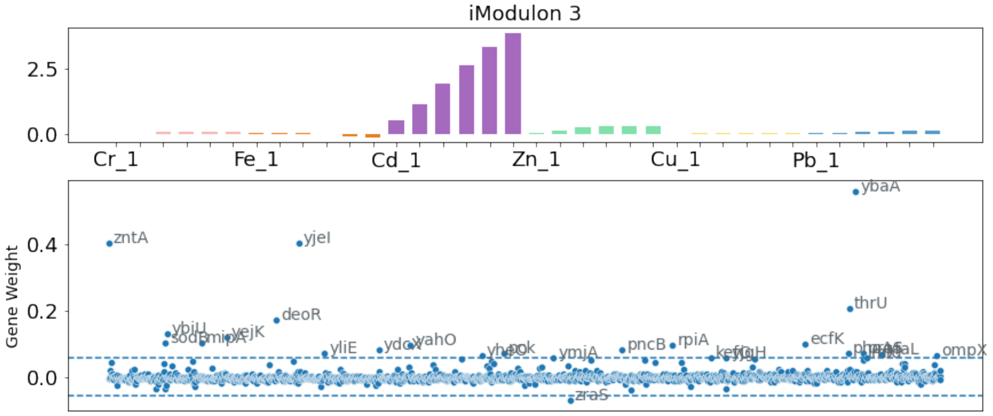

Fig S4|ICA results from running the algorithm on the induction window time points. Top: Activation profile plot of iModulon 3. Bottom: Genes weights above threshold for IModulon 3.

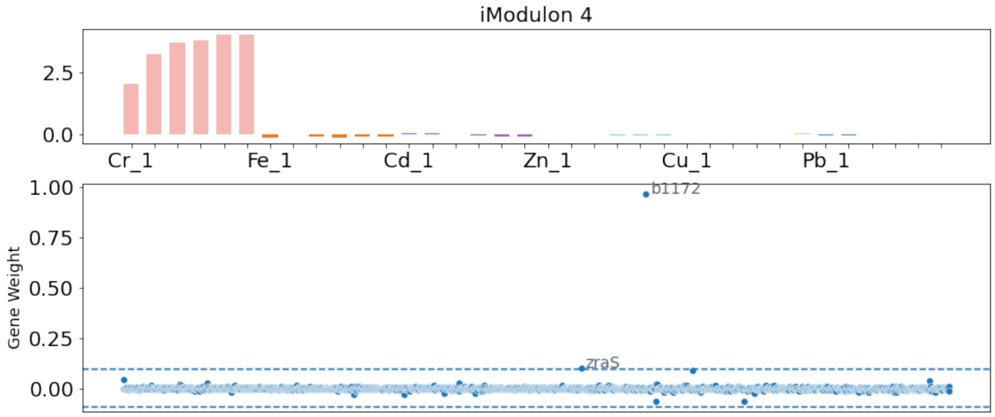

Fig S5|ICA results from running the algorithm on the induction window time points. Top: Activation profile plot of iModulon 4. Bottom: Genes weights above threshold for IModulon 4.

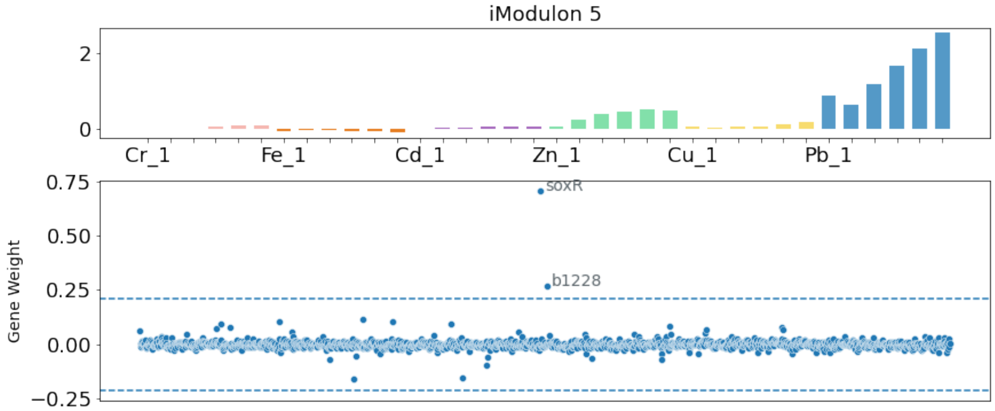

Fig S6|ICA results from running the algorithm on the induction window time points. Top: Activation profile plot of iModulon 5. Bottom: Genes weights above threshold for IModulon 5.

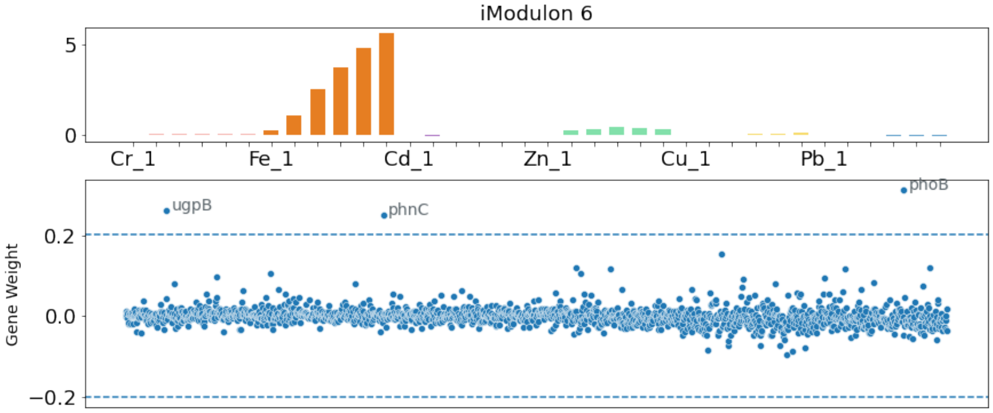

Fig S7|ICA results from running the algorithm on the induction window time points. Top: Activation profile plot of iModulon 6. Bottom: Genes weights above threshold for IModulon 6.

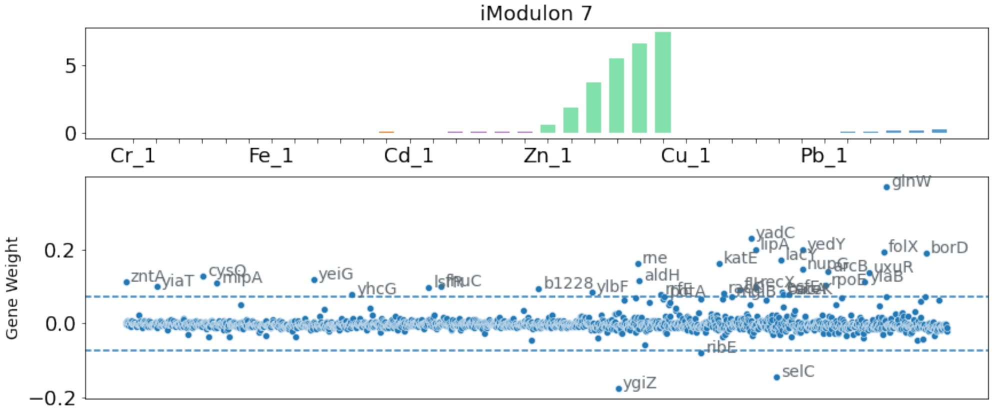

Fig S8|ICA results from running the algorithm on the induction window time points. Top: Activation profile plot of iModulon 7. Bottom: Genes weights above threshold for IModulon 7.

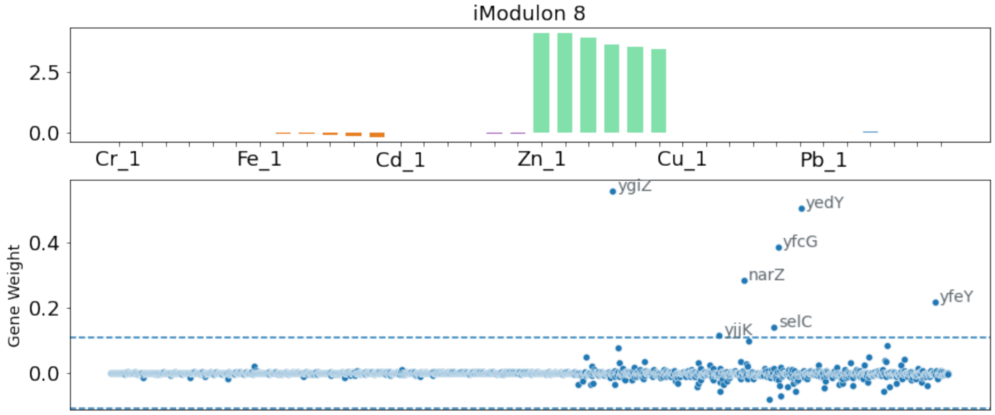

Fig S9|ICA results from running the algorithm on the induction window time points. Top: Activation profile plot of iModulon 8. Bottom: Genes weights above threshold for IModulon 8.

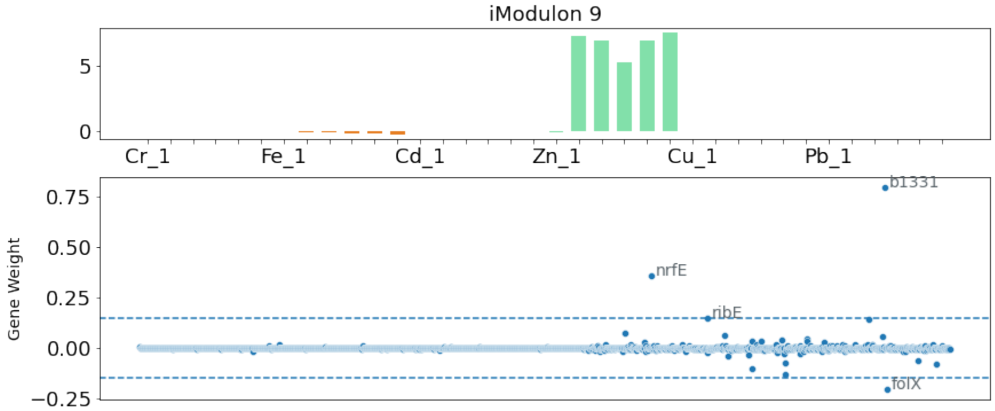

Fig S10|ICA results from running the algorithm on the induction window time points. Top: Activation profile plot of iModulon 9. Bottom: Genes weights above threshold for IModulon 9.

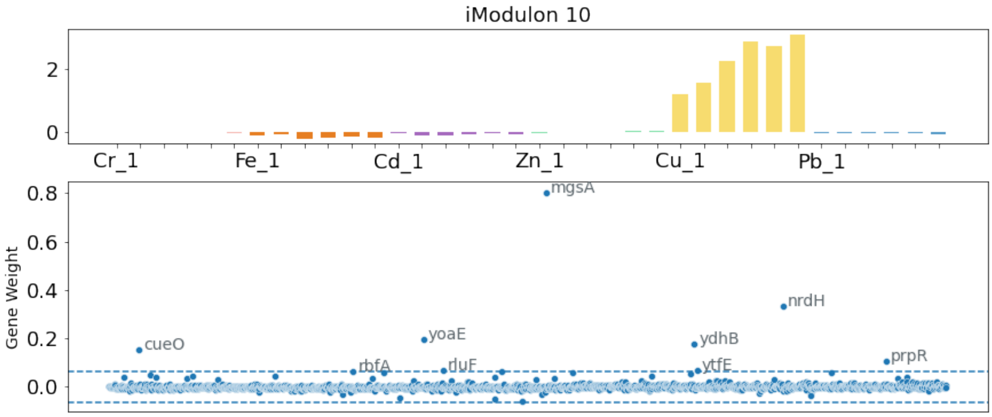

Fig S11|ICA results from running the algorithm on the induction window time points. Top: Activation profile plot of iModulon 10. Bottom: Genes weights above threshold for IModulon 10.

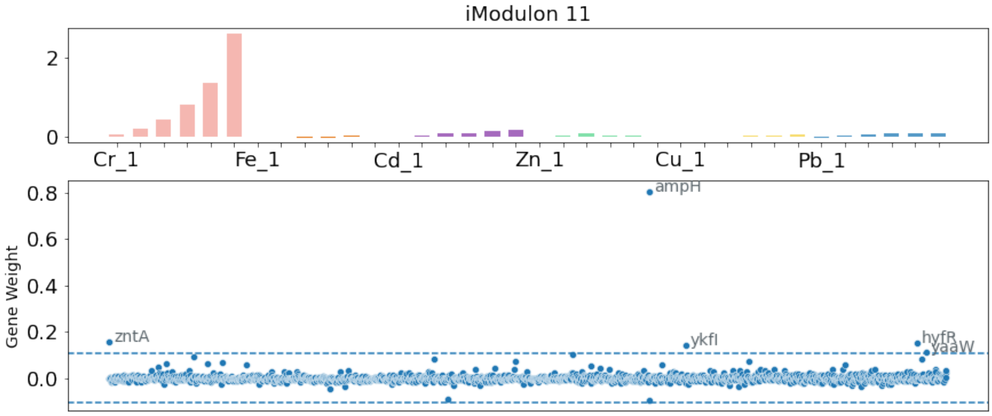

Fig S12|ICA results from running the algorithm on the induction window time points. Top: Activation profile plot of iModulon 11. Bottom: Genes weights above threshold for IModulon 11.

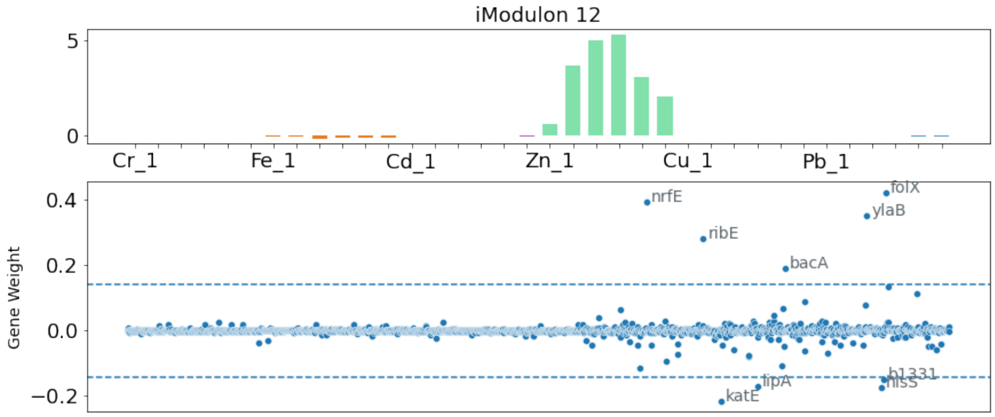

Fig S13|ICA results from running the algorithm on the induction window time points. Top: Activation profile plot of iModulon 12. Bottom: Genes weights above threshold for IModulon 12.

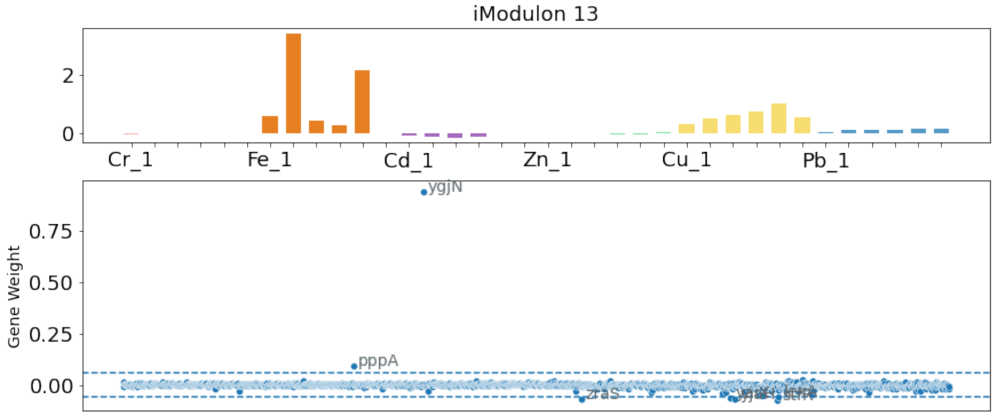

Fig S14|ICA results from running the algorithm on the induction window time points. Top: Activation profile plot of iModulon 13. Bottom: Genes weights above threshold for IModulon 13.

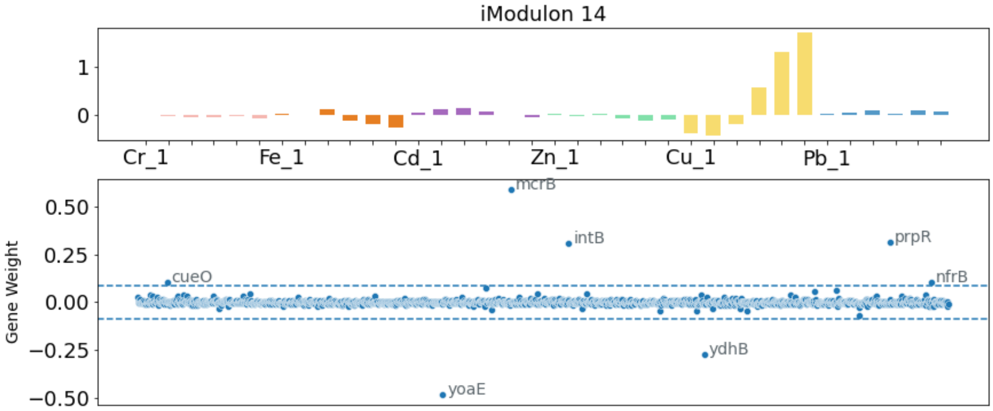

Fig S15|ICA results from running the algorithm on the induction window time points. Top: Activation profile plot of iModulon 14. Bottom: Genes weights above threshold for IModulon 14.

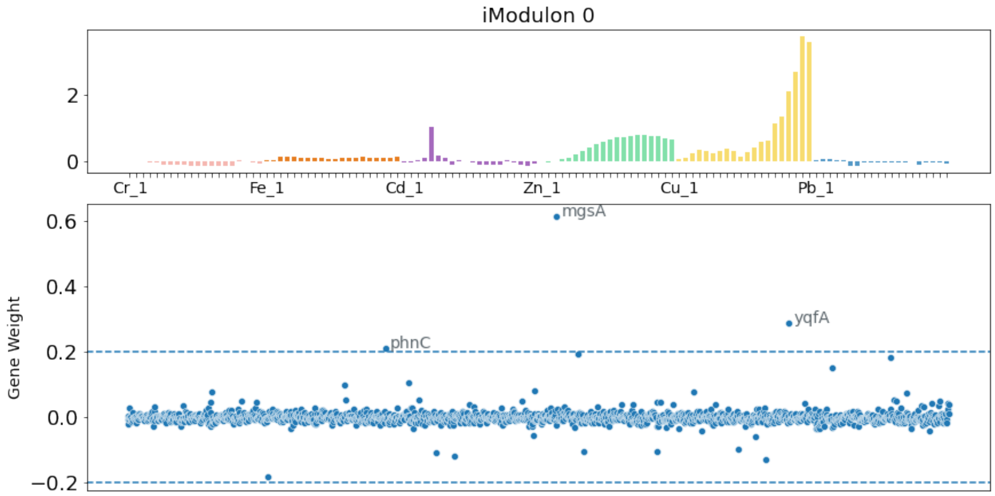

Fig S16|ICA results from running the algorithm on the data post induction. Top: Activation profile plot of iModulon 0. Bottom: Genes weights above threshold for IModulon 0.

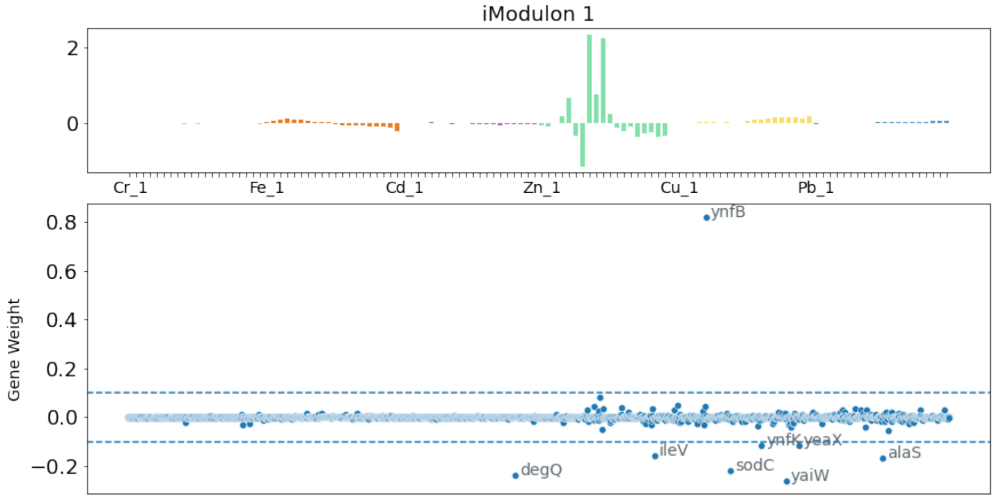

Fig S17|ICA results from running the algorithm on the data post induction. Top: Activation profile plot of iModulon 1. Bottom: Genes weights above threshold for IModulon 1.

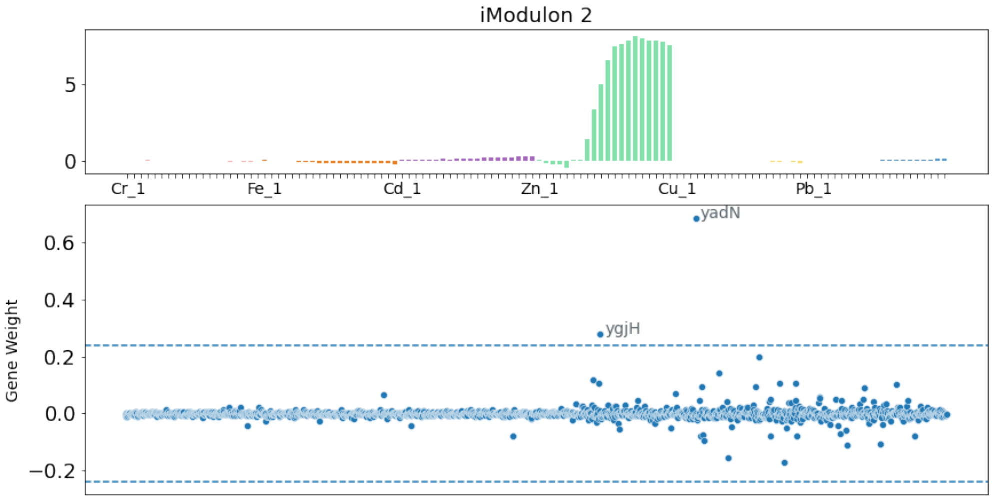

Fig S18|ICA results from running the algorithm on the data post induction. Top: Activation profile plot of iModulon 2. Bottom: Genes weights above threshold for IModulon 2.

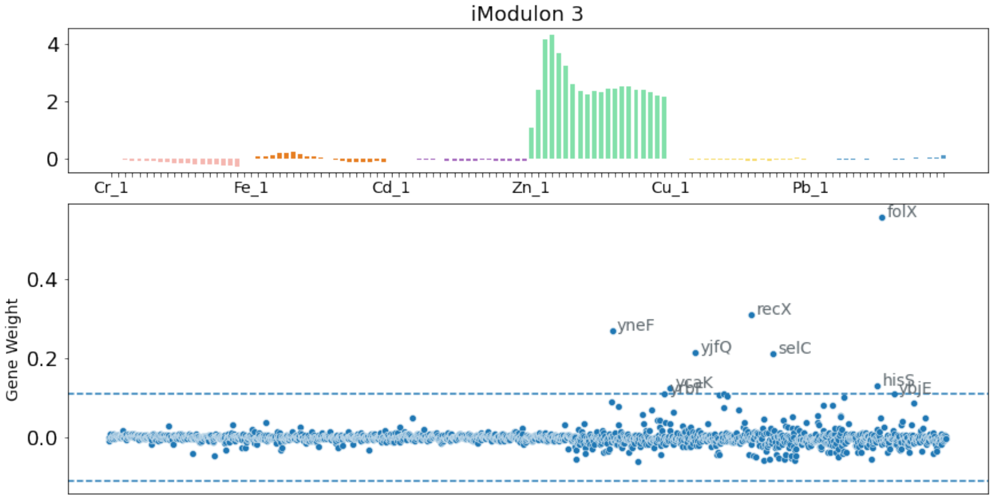

Fig S19|ICA results from running the algorithm on the data post induction. Top: Activation profile plot of iModulon 3. Bottom: Genes weights above threshold for IModulon 3.

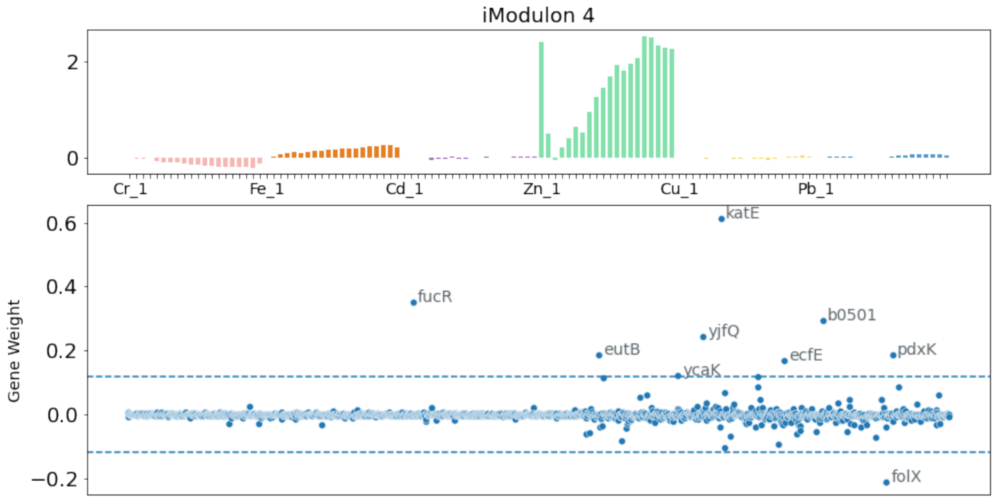

Fig S20|ICA results from running the algorithm on the data post induction. Top: Activation profile plot of iModulon 4. Bottom: Genes weights above threshold for IModulon 4.

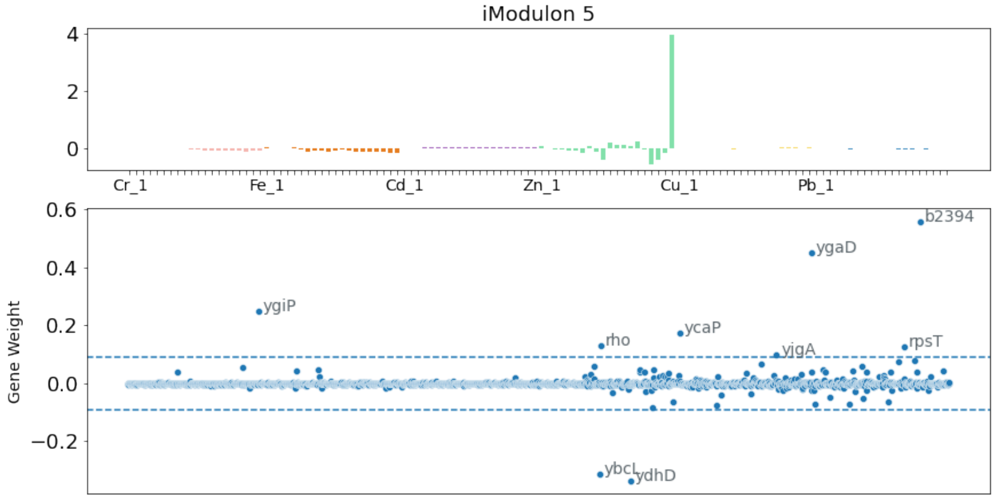

Fig S21|ICA results from running the algorithm on the data post induction. Top: Activation profile plot of iModulon 5. Bottom: Genes weights above threshold for IModulon 5.

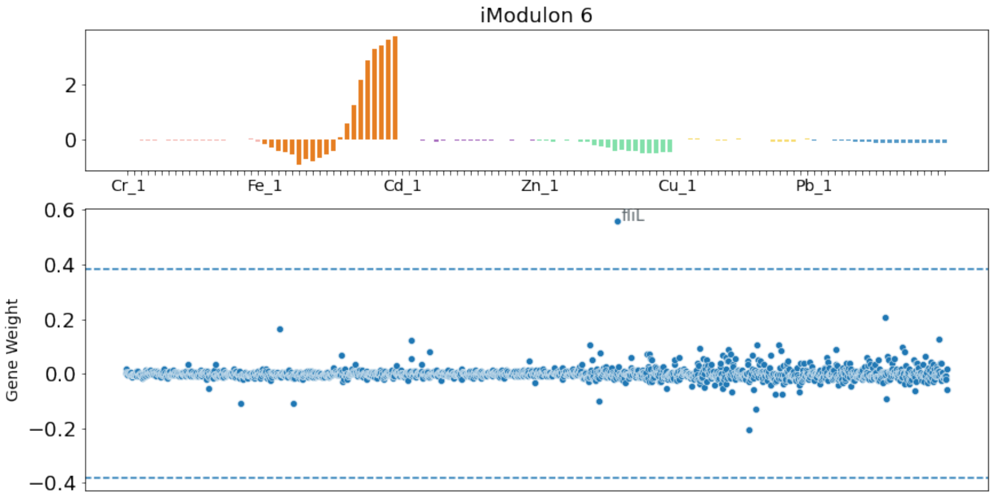

Fig S22|ICA results from running the algorithm on the data post induction. Top: Activation profile plot of iModulon 6. Bottom: Genes weights above threshold for IModulon 6.

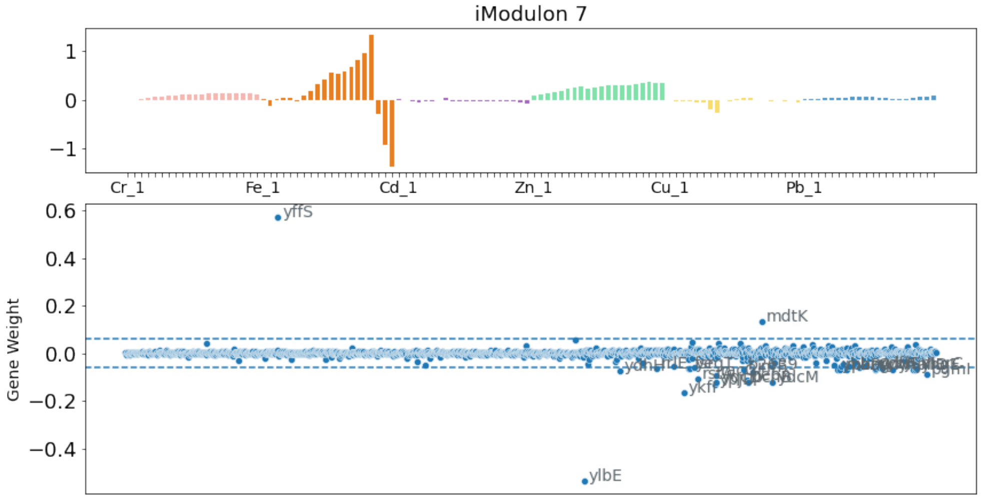

Fig S23|ICA results from running the algorithm on the data post induction. Top: Activation profile plot of iModulon 7. Bottom: Genes weights above threshold for IModulon 7.

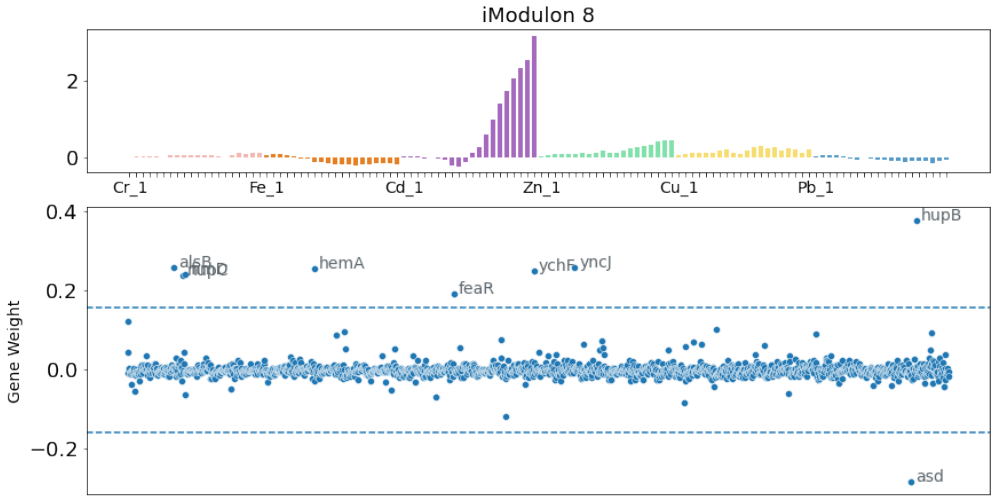

Fig S24|ICA results from running the algorithm on the data post induction. Top: Activation profile plot of iModulon 8. Bottom: Genes weights above threshold for IModulon 8.

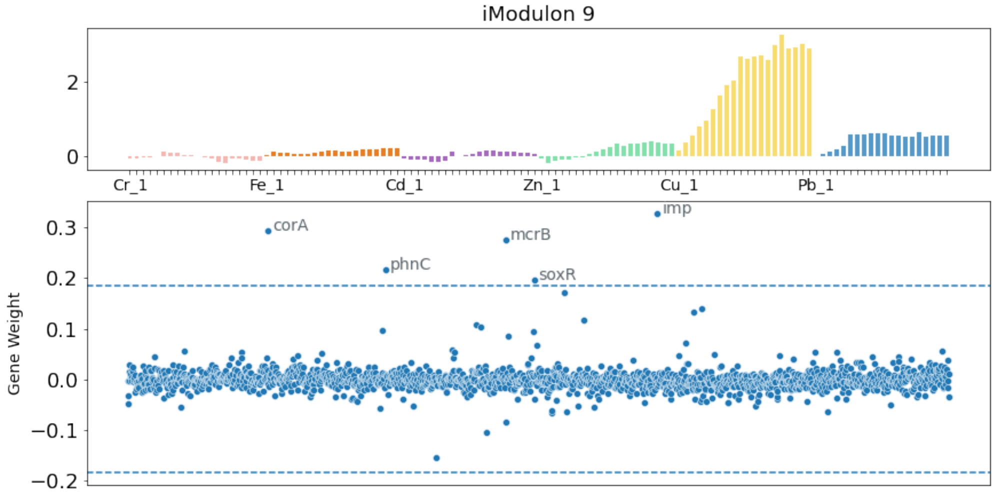

Fig S25|ICA results from running the algorithm on the data post induction. Top: Activation profile plot of iModulon 9. Bottom: Genes weights above threshold for IModulon 9.

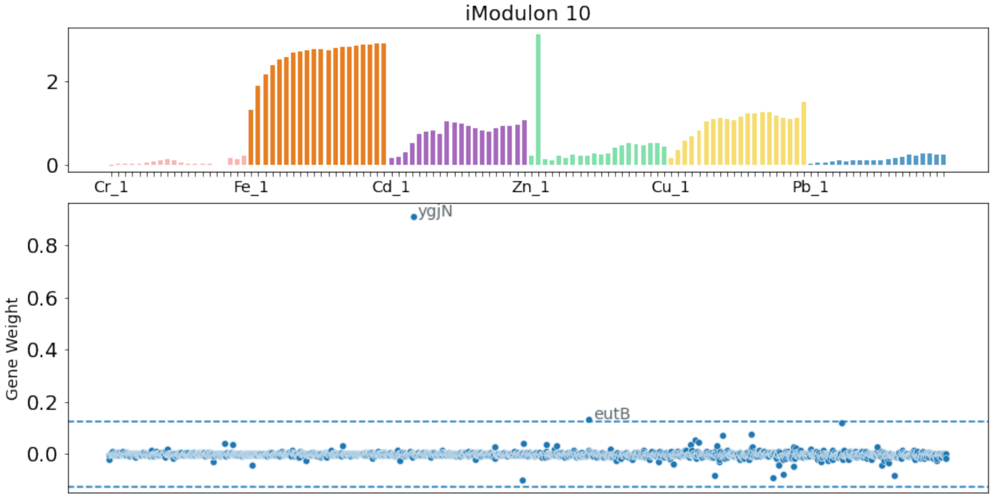

Fig S26|ICA results from running the algorithm on the data post induction. Top: Activation profile plot of iModulon 10. Bottom: Genes weights above threshold for IModulon 10.

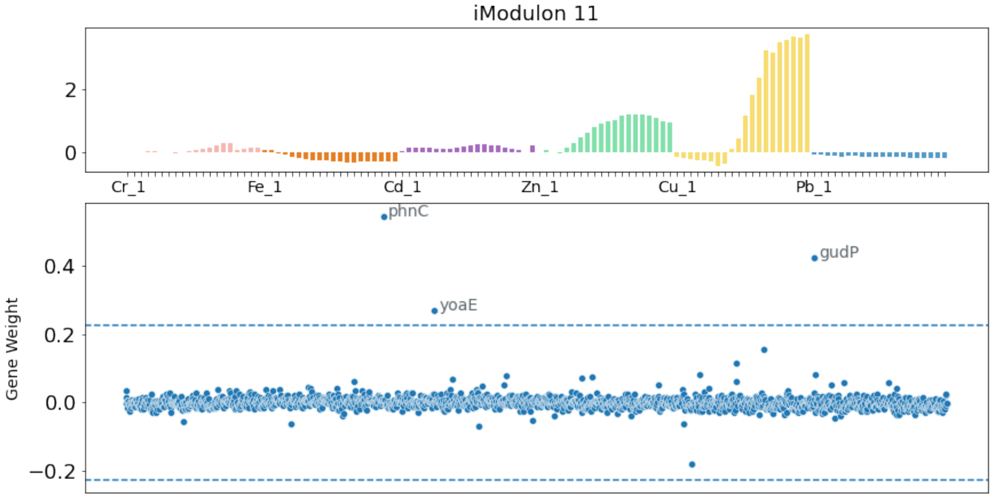

Fig S27|ICA results from running the algorithm on the data post induction. Top: Activation profile plot of iModulon 11. Bottom: Genes weights above threshold for IModulon 11.

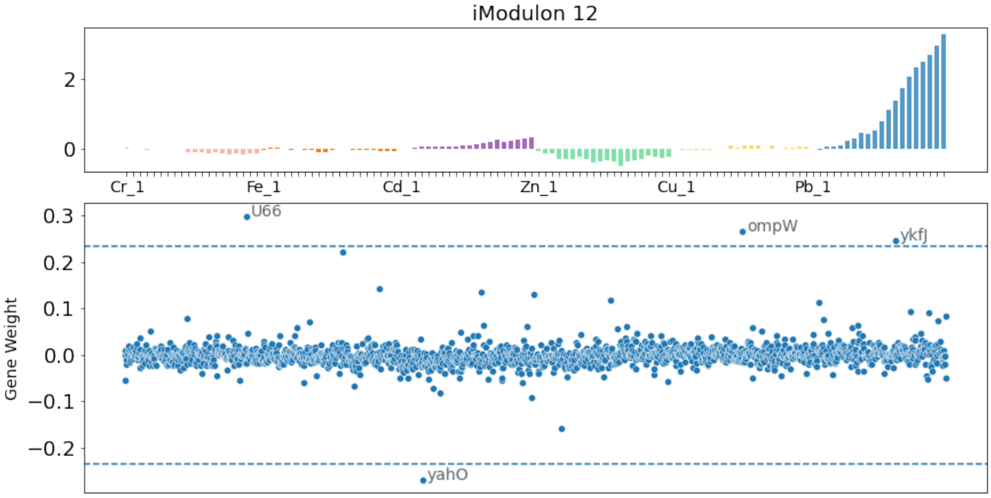

Fig S28|ICA results from running the algorithm on the data post induction. Top: Activation profile plot of iModulon 12. Bottom: Genes weights above threshold for IModulon 12.

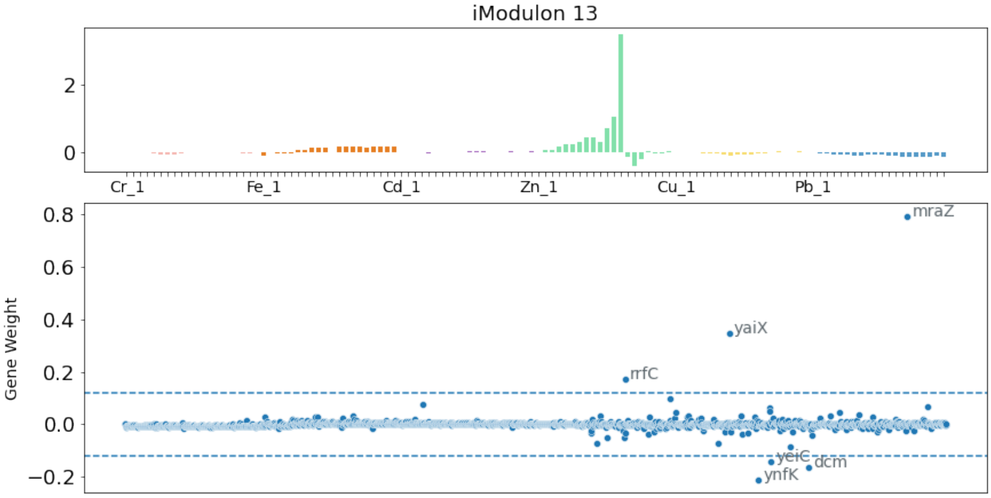

Fig S29|ICA results from running the algorithm on the data post induction. Top: Activation profile plot of iModulon 13. Bottom: Genes weights above threshold for IModulon 13.

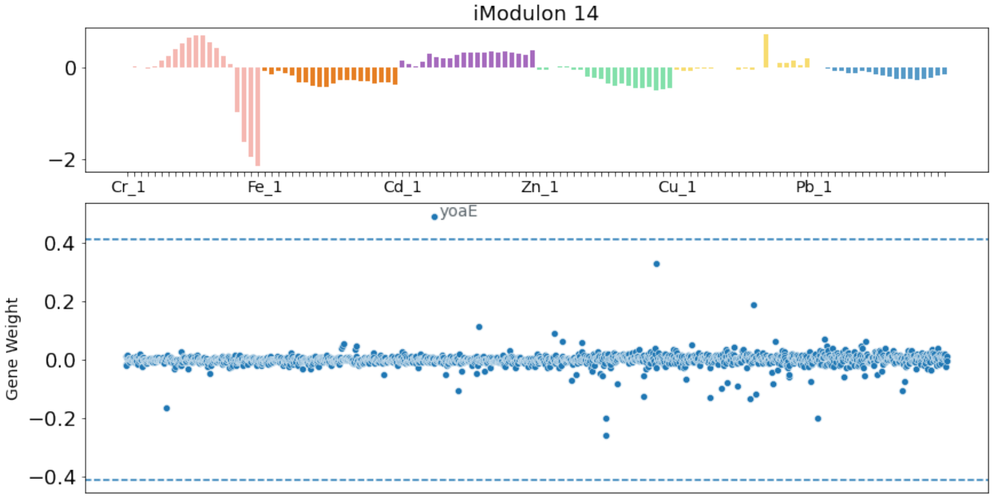

Fig S30|ICA results from running the algorithm on the data post induction. Top: Activation profile plot of iModulon 14. Bottom: Genes weights above threshold for IModulon 14.

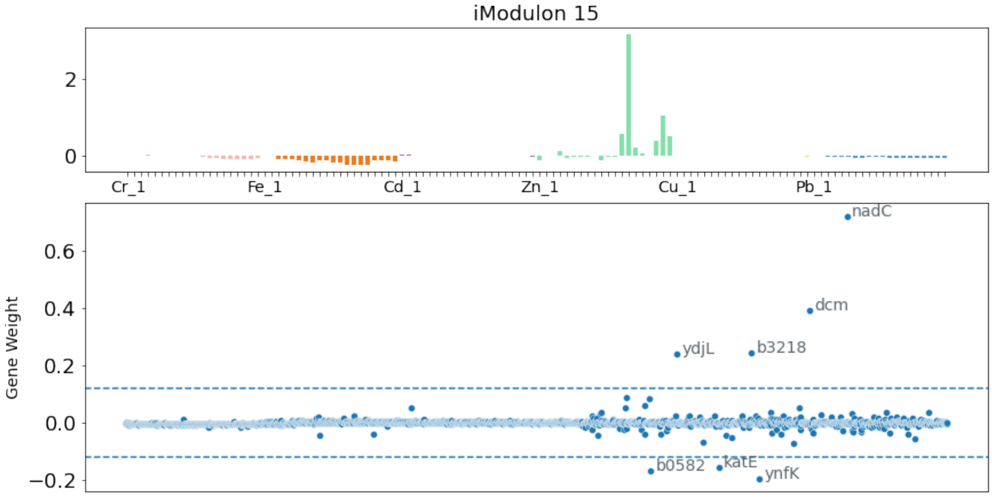

Fig S31|ICA results from running the algorithm on the data post induction. Top: Activation profile plot of iModulon 15. Bottom: Genes weights above threshold for IModulon 15.

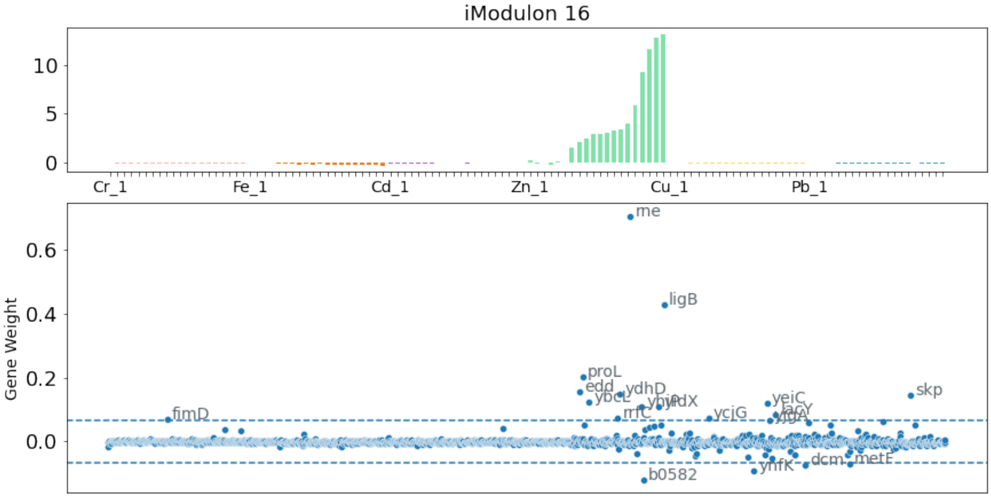

Fig S32|ICA results from running the algorithm on the data post induction. Top: Activation profile plot of iModulon 16. Bottom: Genes weights above threshold for IModulon 16.

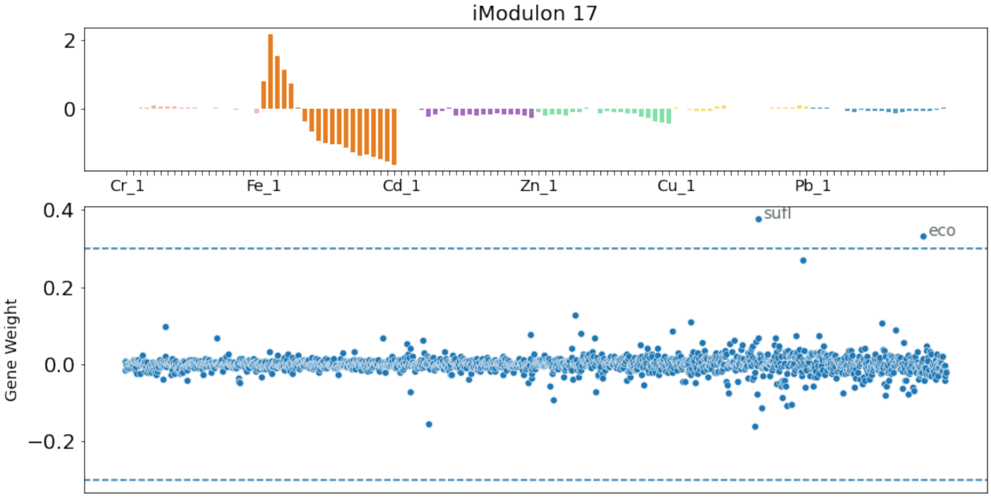

Fig S33|ICA results from running the algorithm on the data post induction. Top: Activation profile plot of iModulon 17. Bottom: Genes weights above threshold for IModulon 17.

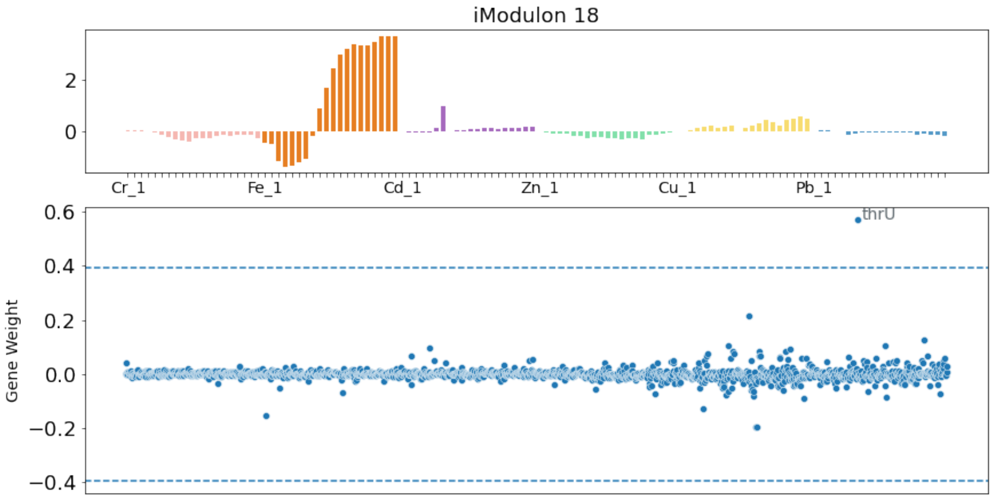

Fig S34|ICA results from running the algorithm on the data post induction. Top: Activation profile plot of iModulon 18. Bottom: Genes weights above threshold for IModulon 18.

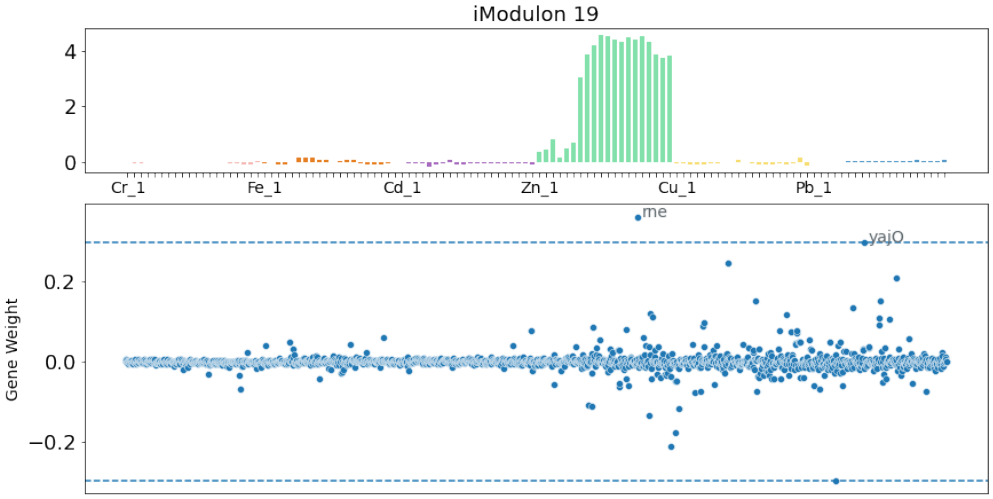

Fig S35|ICA results from running the algorithm on the data post induction. Top: Activation profile plot of iModulon 19. Bottom: Genes weights above threshold for IModulon 19.

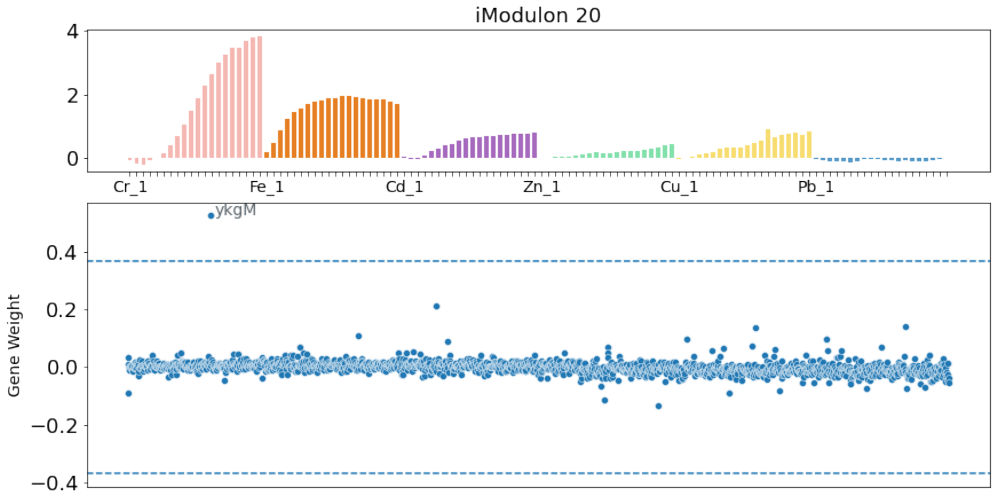

Fig S36|ICA results from running the algorithm on the data post induction. Top: Activation profile plot of iModulon 20. Bottom: Genes weights above threshold for IModulon 20.

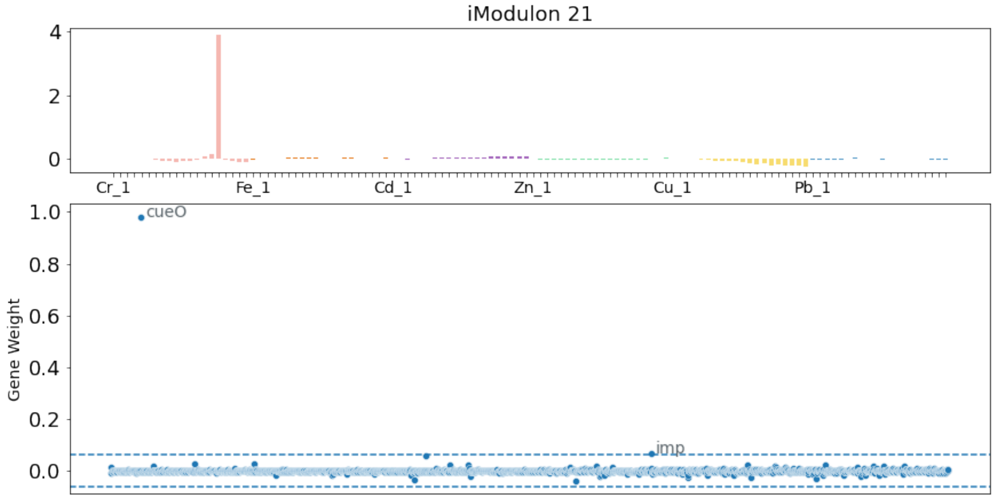

Fig S37|ICA results from running the algorithm on the data post induction. Top: Activation profile plot of iModulon 21. Bottom: Genes weights above threshold for IModulon 21.

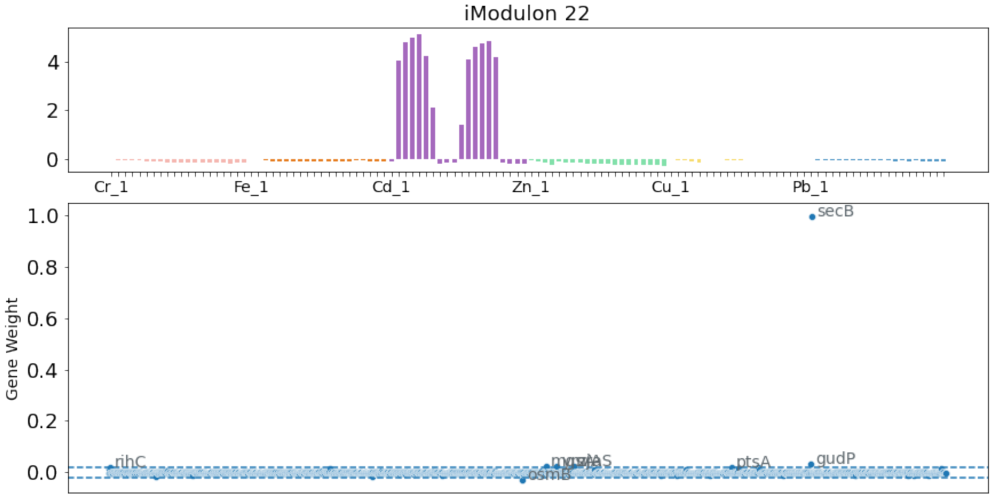

Fig S38|ICA results from running the algorithm on the data post induction. Top: Activation profile plot of iModulon 22. Bottom: Genes weights above threshold for IModulon 22.

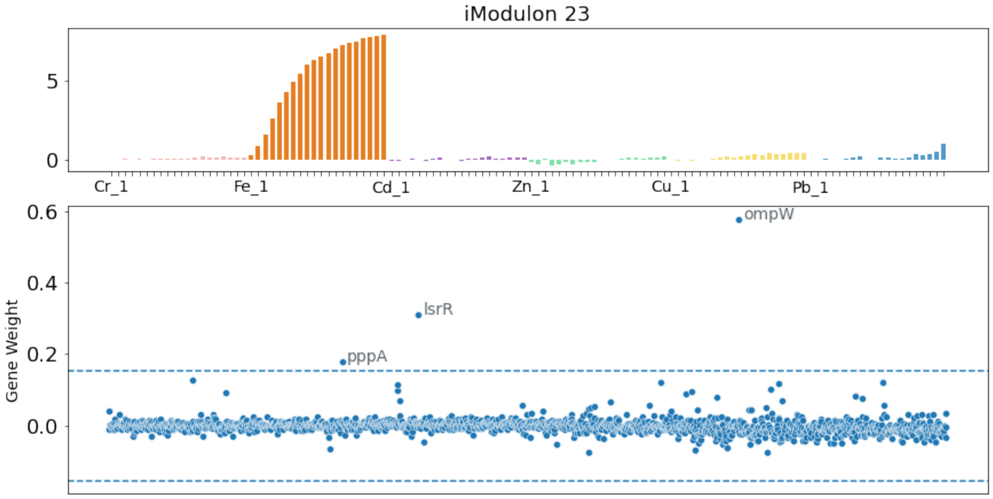

Fig S39|ICA results from running the algorithm on the data post induction. Top: Activation profile plot of iModulon 23. Bottom: Genes weights above threshold for IModulon 23.

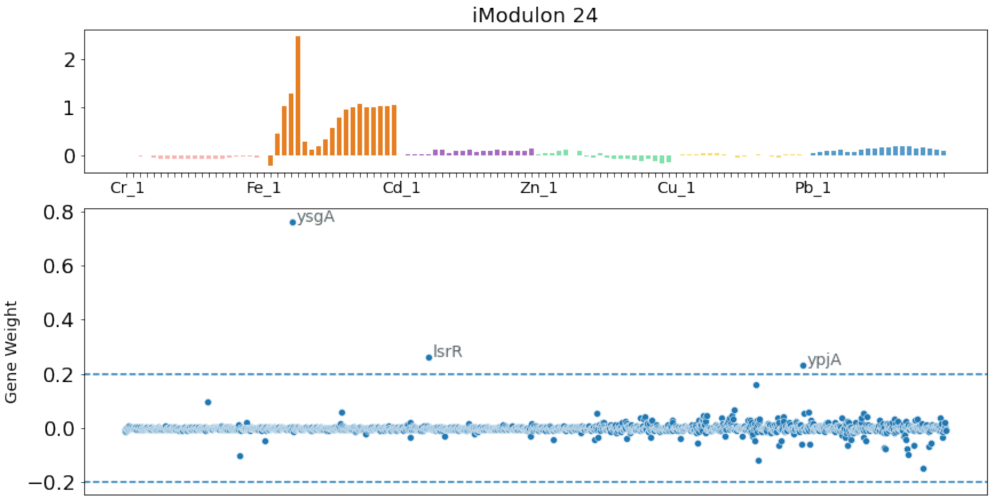

Fig S40|ICA results from running the algorithm on the data post induction. Top: Activation profile plot of iModulon 24. Bottom: Genes weights above threshold for IModulon 24.

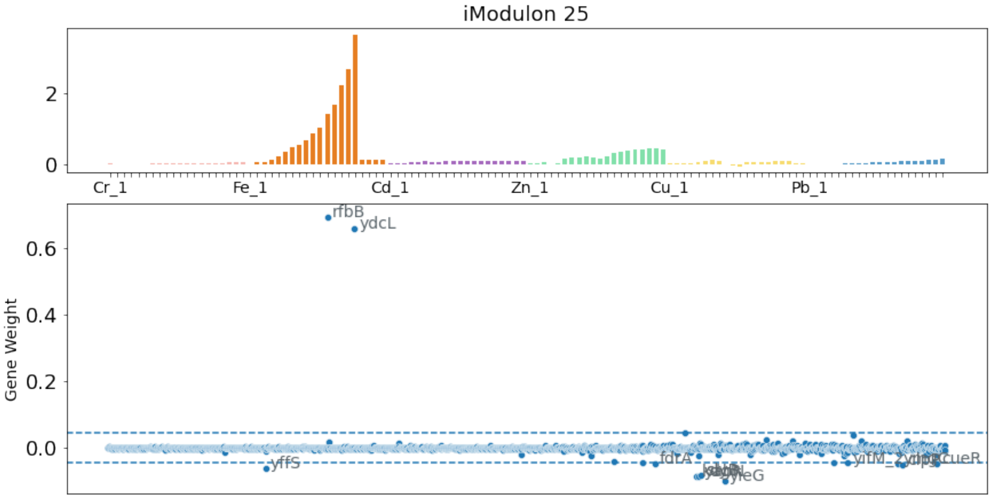

Fig S41|ICA results from running the algorithm on the data post induction. Top: Activation profile plot of iModulon 25. Bottom: Genes weights above threshold for IModulon 25.

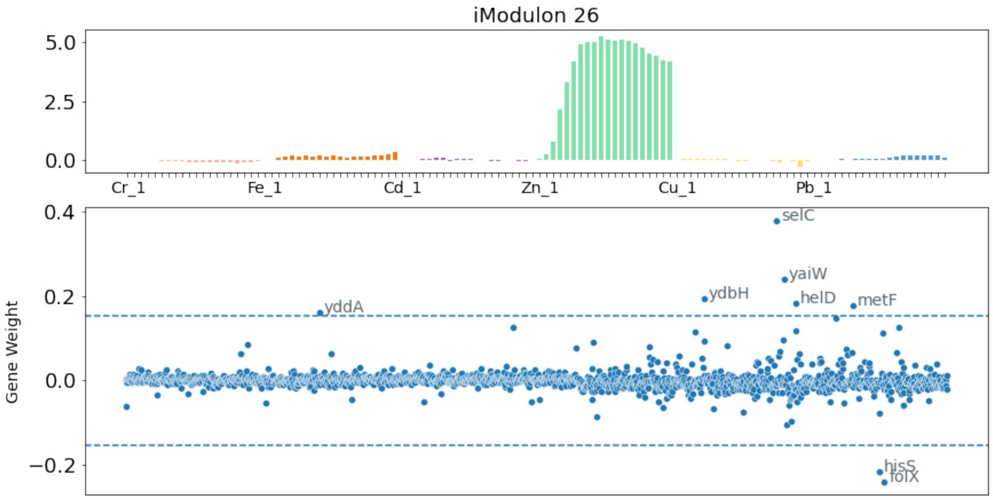

Fig S42|ICA results from running the algorithm on the data post induction. Top: Activation profile plot of iModulon 26. Bottom: Genes weights above threshold for IModulon 26.

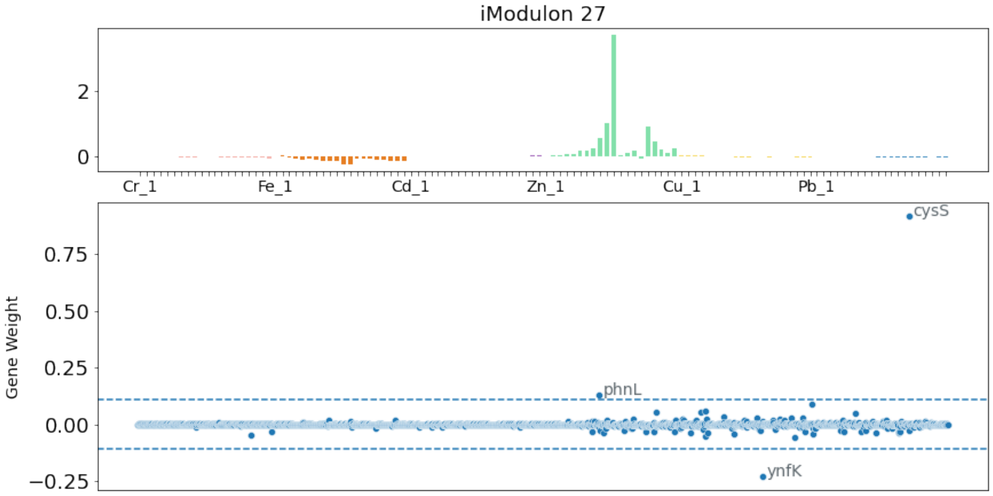

Fig S43|ICA results from running the algorithm on the data post induction. Top: Activation profile plot of iModulon 27. Bottom: Genes weights above threshold for IModulon 27.

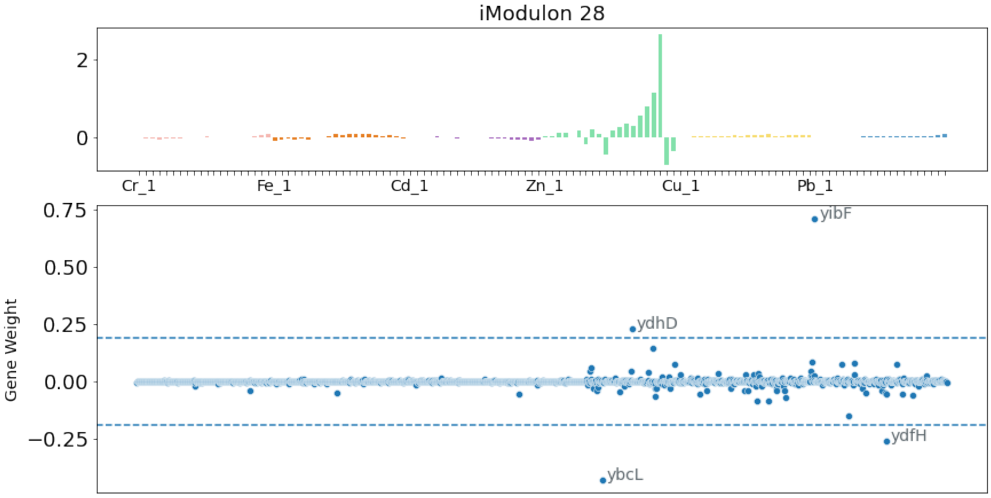

Fig S44|ICA results from running the algorithm on the data post induction. Top: Activation profile plot of iModulon 28. Bottom: Genes weights above threshold for IModulon 28.

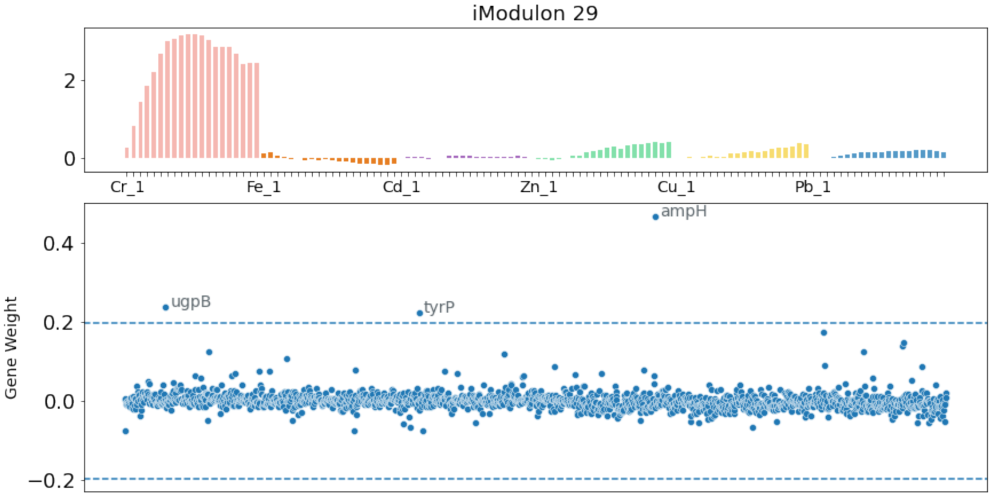

Fig S45|ICA results from running the algorithm on the data post induction. Top: Activation profile plot of iModulon 29. Bottom: Genes weights above threshold for IModulon 29.

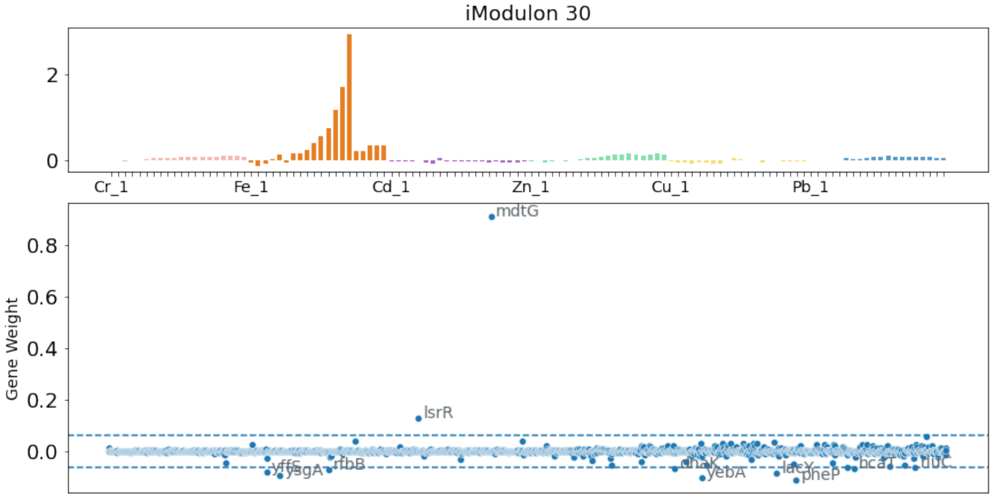

Fig S46|ICA results from running the algorithm on the data post induction. Top: Activation profile plot of iModulon 30. Bottom: Genes weights above threshold for IModulon 30.

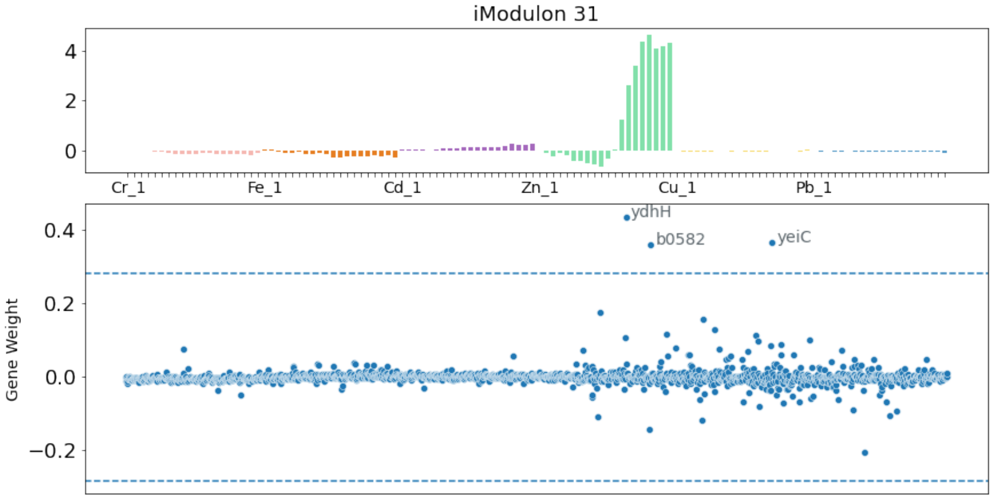

Fig S47|ICA results from running the algorithm on the data post induction. Top: Activation profile plot of iModulon 31. Bottom: Genes weights above threshold for IModulon 31.

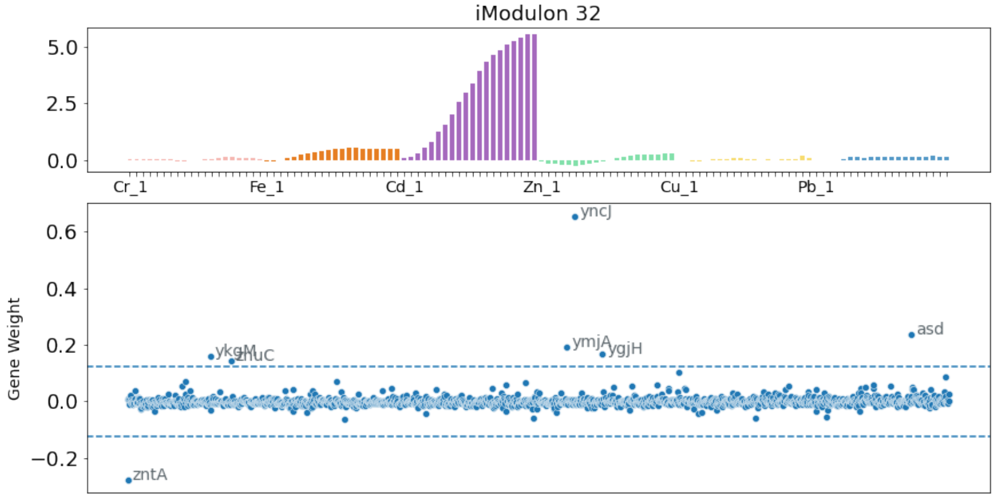

Fig S48|ICA results from running the algorithm on the data post induction. Top: Activation profile plot of iModulon 32. Bottom: Genes weights above threshold for IModulon 32.

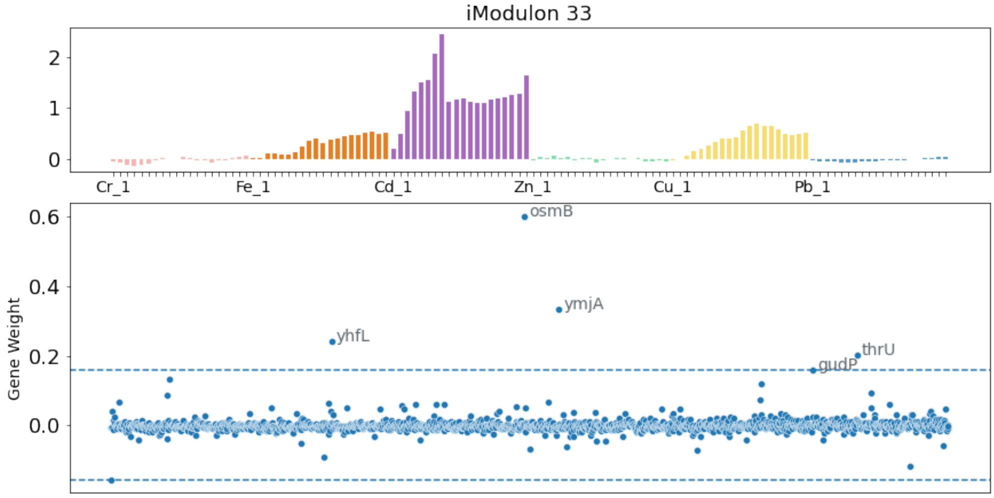

Fig S49|ICA results from running the algorithm on the data post induction. Top: Activation profile plot of iModulon 33. Bottom: Genes weights above threshold for IModulon 33.

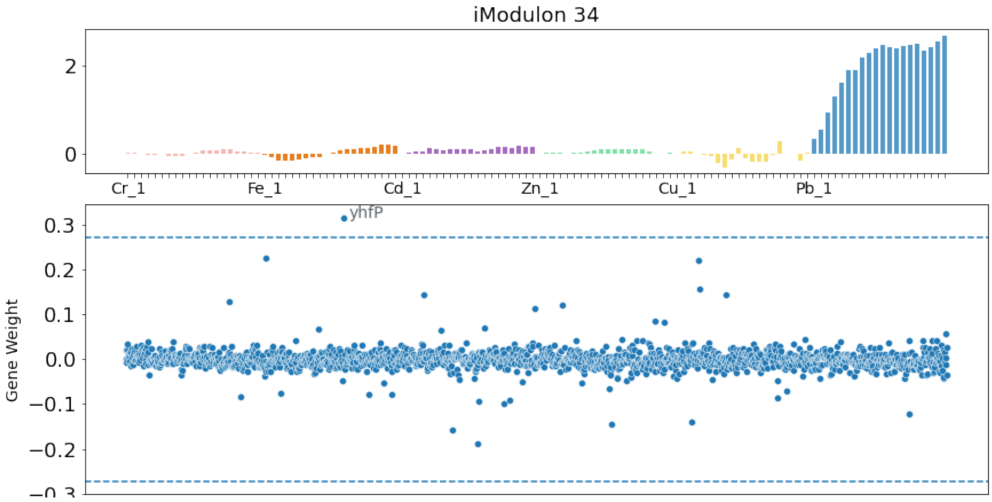

Fig S50|ICA results from running the algorithm on the data post induction. Top: Activation profile plot of iModulon 34. Bottom: Genes weights above threshold for IModulon 34.

Heavy metal	Concentration data (uM)	MIC (mM)
Zn	0.765	2
Cr	5	1.7
Cu	2	1.57
Pb	0.14	1.92
Cd	0.04	2.66
Fe	5	Na

Fig S51|Table with the concentrations of the heavy metals used in this study as well as the MIC reported in the literature. Iron MIC is not reported given that it's an essential compound for the cells.

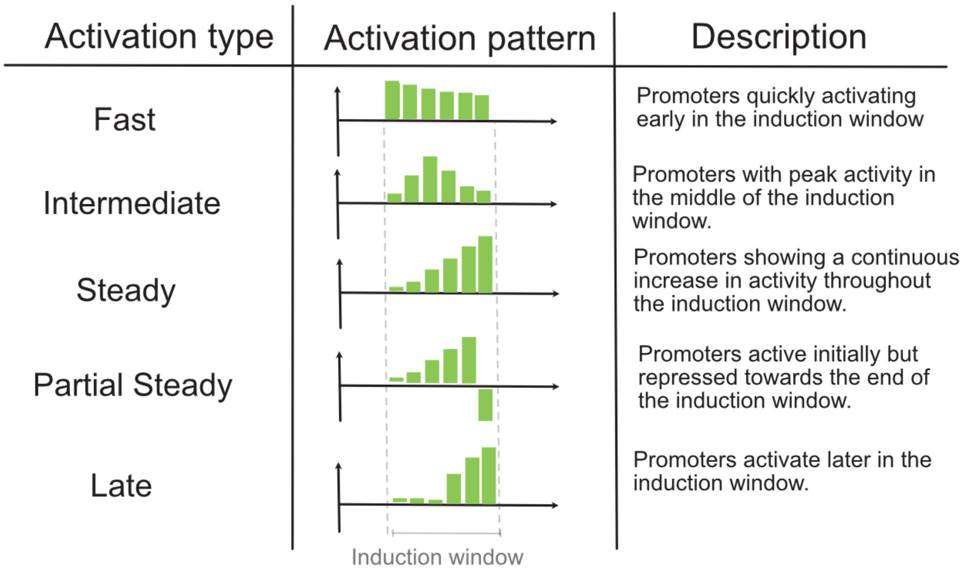

Fig S52|Classification of most frequent promoter activation patterns observed .

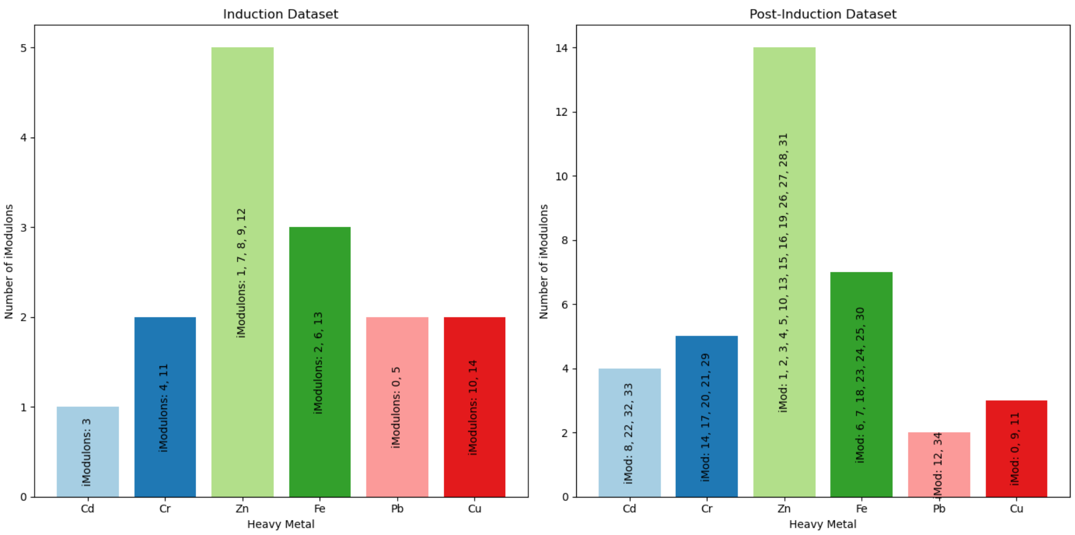

Fig S53| Overall identification of iModulons based on the heavy metal with highest aggregate coefficient in the activation window.

iModulon	Enriched_Metal	center_of_mass	highest_peak	shape_classification
0	Pb	6.00937124004475	6	Mixed Activation
1	Zn	1.9586905603404643	5	Steady Activation
2	Fe	2.8858609181047212	4	Intermediate Activation
3	Cd	3.400290595224472	6	Steady Activation
4	Cr	2.797106658835455	5	Steady Activation
5	Pb	3.226218187718445	6	Steady Activation
6	Fe	3.577980624456813	6	Steady Activation
7	Zn	3.485144831724121	6	Steady Activation
8	Zn	2.382074032444398	2	Fast Activation
9	Zn	3.0220339595067416	6	Steady Activation
10	Cu	2.994608670476212	6	Steady Activation
11	Cr	4.017869426039383	6	Steady Activation
12	Zn	2.6424086644095337	4	Intermediate Activation
13	Fe	1.9954508079486497	2	Fast Activation
14	Cu	5.714600247227268	6	Mixed Activation

Fig S54| Classification of each iModulon described in Figure 2 and 3 according to the shape of the activity coefficients within the induction window of heavy metal enriched.
