# Supplementary material for: High-resolution temporal profiling of E. coli transcriptional response
Source: Nat Commun. 2023 Nov 22;14:7606. doi: 10.1038/s41467-023-43173-7 (PMC10665441; doi:10.1038/s41467-023-43173-7)
Supplement: Supplementary file 3 — Description of Additional Supplementary Files [file 41467_2023_43173_MOESM3_ESM.pdf]

Description of Additional Supplementary Files:

Supplementary Dataset 1: Data matrix X (Figure 2,3) composed by 1,805 rows representing the different promoters and 36 columns representing the log2 of the fold change for each time point during the six heavy metal inductions, for a total of 36 (6 different heavy metals multiplied by 6 different induction time windows).

Supplementary Dataset 2: Gene coefficient matrix M (Figure 2,3) obtained by applying ICA on Dataset 1.

Supplementary Dataset 3: Activity coefficient matrix A (Figure 2,3) obtained by applying ICA on Dataset 1.

Supplementary Dataset 4: Data matrix X (for Figure 4) built using the time points recorded during the recovery period post-induction. The matrix is composed by 1,805 rows representing the different promoters and columns representing the log2 of the fold change for each time point during the six heavy metal inductions, for a total of 180 (6 different heavy metals multiplied by 20 time points).

Supplementary Dataset 5: Gene coefficient matrix M (Figure 4) obtained by applying ICA on Dataset 4.

Supplementary Dataset 6: Activity coefficient matrix A (Figure 4) obtained by applying ICA on Dataset 4.
